# Successfully treating hand primary tuberculous synovitis by synovectomy combined antituberculous therapy: A case report

**DOI:** 10.1097/MD.0000000000009938

**Published:** 2018-02-23

**Authors:** Tao Wang, Gang Zhao, Yong-Jun Rui, Jing-Yi Mi

**Affiliations:** Department of Hand Surgery, Wuxi NO.9 People's Hospital Affiliated to Soochow University, Wuxi, China.

**Keywords:** hand, *Mycobacterium tuberculosis*, radical synovectomy, synovitis, wrist

## Abstract

**Rationale:**

Primary tuberculous infection in hand and wrist is a rare disease. Few articles reported on hand primary tuberculous synovitis.

**Patient concerns:**

A 68-year-old Chinese male, without history of tuberculosis (TB), had complained of pain and swelling in right palm and little finger for 3 months. Patient came to our hospital on 9th Oct 2016. X-ray just showed soft tissue swelling in little finger. Magnetic resonance imaging (MRI) showed synovitis around flexor tendon of little finger, volar palm, and carpal tunnel. Notably, it also implied nodular images in little finger sizing 5 mm × 11 mm. Laboratory tests revealed C-reactive protein (CRP): 22 mg/L, erythrocyte sedimentation rate (ESR): 49 mm/h, and white blood cells (WBC): 11.8 × 10^9^/L.

**Diagnoses:**

He was diagnosed with primary hand tuberculous synovitis.

**Interventions:**

The patient received aspiration biopsy in right palm guided by ultrasound on 13rd Oct and pathological examination indicated *Mycobacterium tuberculosis* (MTB) infection. We performed radical synovetomy and collected abnormal tissue for pathological examination on 18th Oct. Finally, result showed MTB infection, which was the same with the result of first pathological examination. Then, this patient received antituberculous treatment.

**Outcomes:**

One year after operation, pain and swelling relieve and no recurrence of the clinical symptoms happened.

**Lessons:**

Primary tuberculous synovitis of hand and wrist is rare, MTB infection should be considered as an infectious agent, especially in developing countries. Radical synovectomy and antituberculous treatment regain a satisfactory outcome.

## 1 Introduction

Hand tuberculous infection is uncommon, especially for hand tuberculous synovitis.^[1]^ Previous articles reported that the incidence of extrapulmonary tuberculosis ranged from 10% to 15%, of which the occurrence of hand tuberculous infection accounted for <1%.^[1–3]^ As we known, extrapulmonary tuberculous infection was secondary to pulmonary tuberculous.^[2]^ Prakash and Mehtani^[4]^ presented a rare case that a teenager suffered from tuberculosis infection of isolated scaphoid and the patient was treated with multidrug chemotherapy. At 2-year follow-up, the condition had no recurrence. Soman et al^[5]^ showed a case that a patient suffered from tuberculosis infection after extra articular fracture of the distal radius treated by internal fixation. The diagnosis for hand tuberculosis is usually achieved with magnetic resonance imaging (MRI) and confirmed by histopathology and tubercular cultures. Here, we show a rare case on hand primary tuberculous synovitis. As far as we know, few reports on this topic.

## 2 Consent

The current study was approved by ethics committee of the Wuxi NO.9 People's Hospital Affiliated to Soochow University. There is no need to obtain informed consent from the patient because all the data were collected and analyzed anonymously. Patient has consent to be the subject of the report.

## 3 Case report

**Figure 1:**
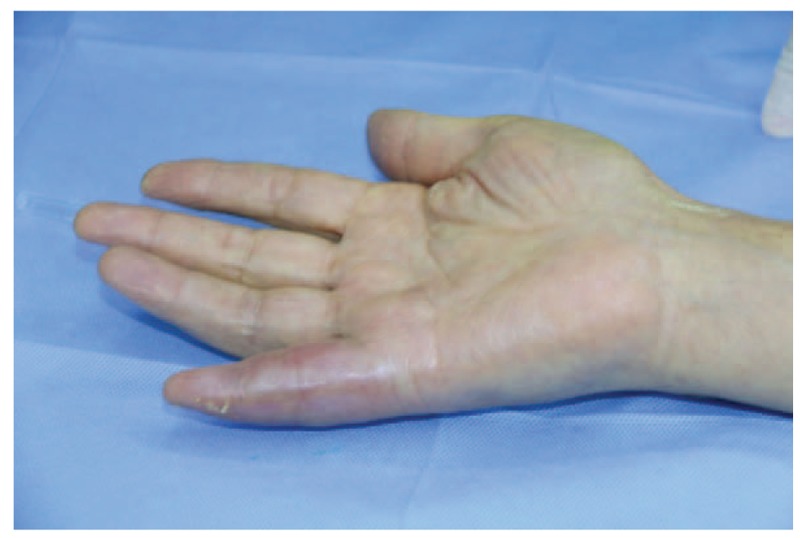
Swelling of right palm and little finger.

**Figure 2:**
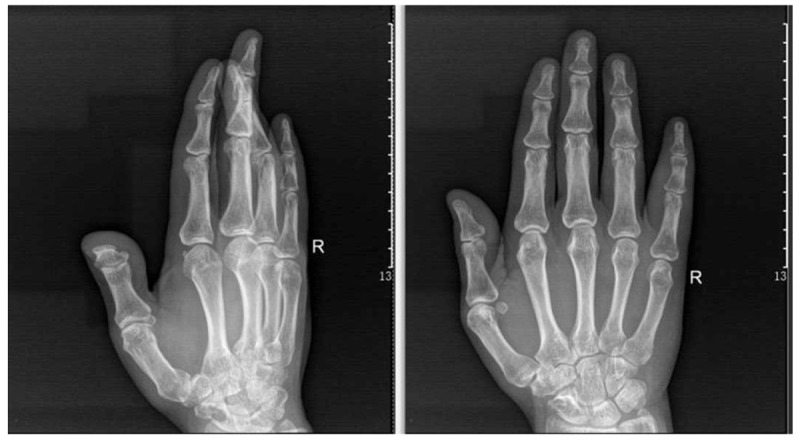
Soft tissue swelling in little finger in x-ray.

**Figure 3:**
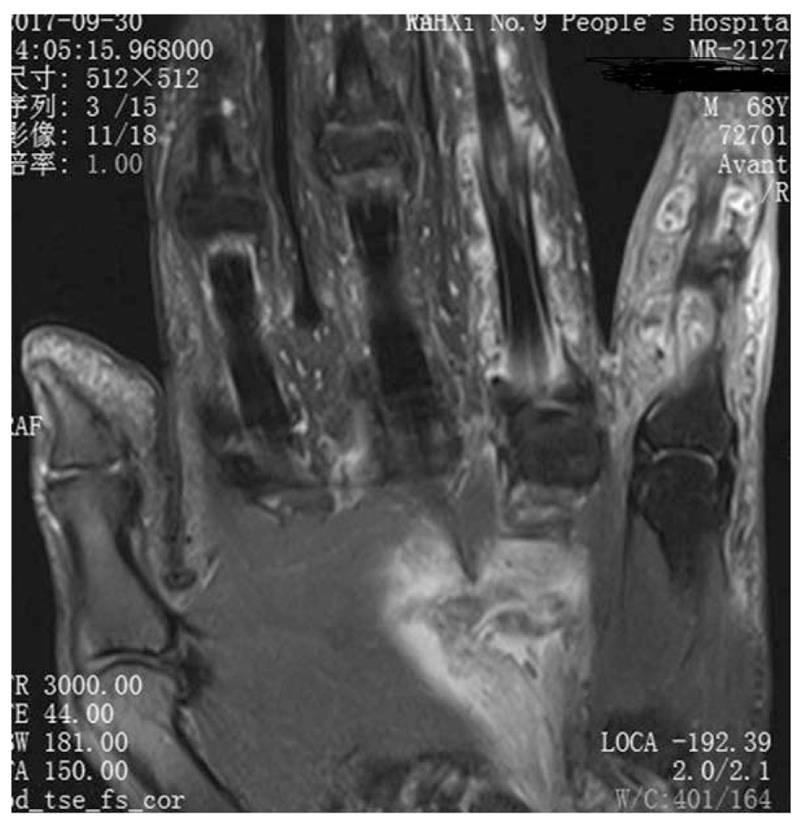
High signal in area of little finger and palm in T2-weighted sequences of MRI. MRI = magnetic resonance imaging.

**Figure 4:**
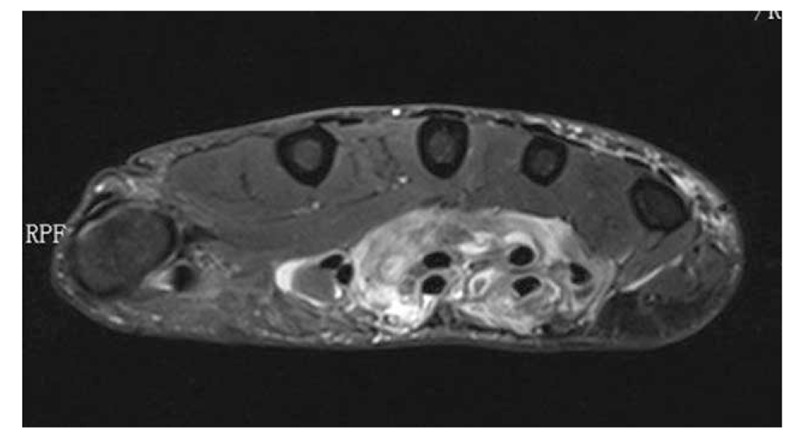
High signal in area of wrist in T2-weighted sequences of MRI. MRI = magnetic resonance imaging.

**Figure 5:**
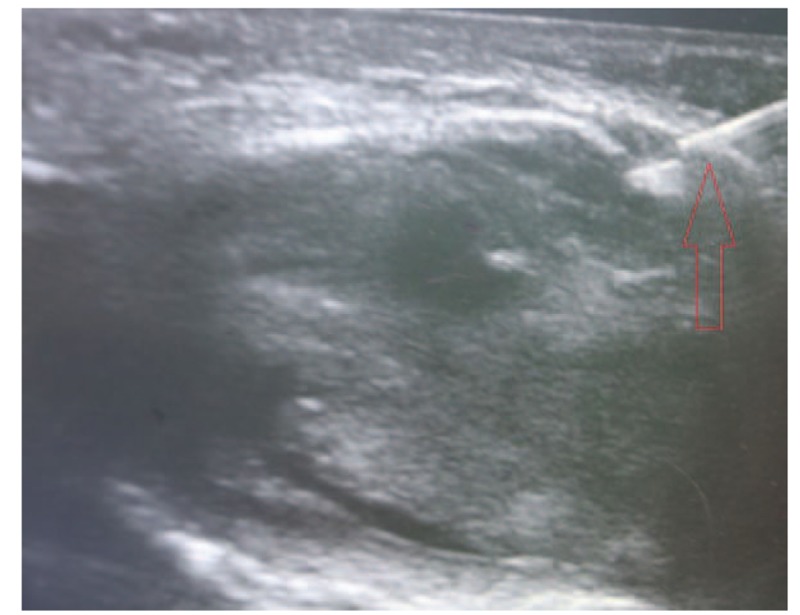
Aspiration biopsy guided by ultrasound.

**Figure 6:**
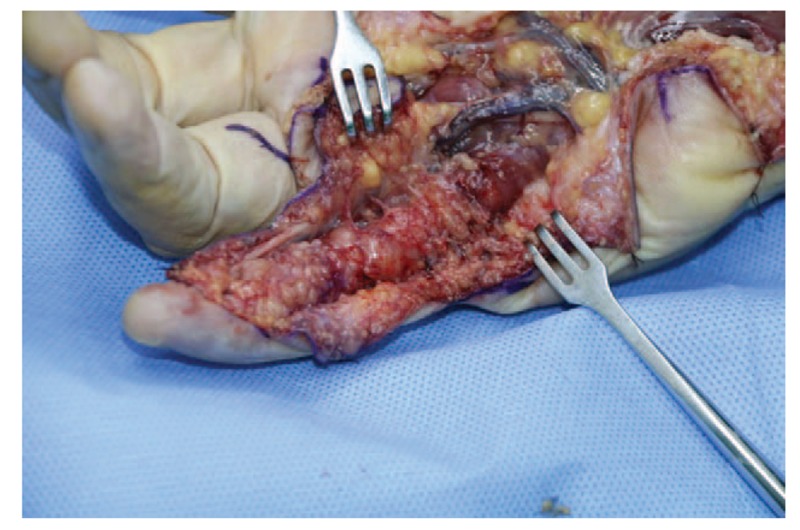
Radical debridement in area of little finger.

**Figure 7:**
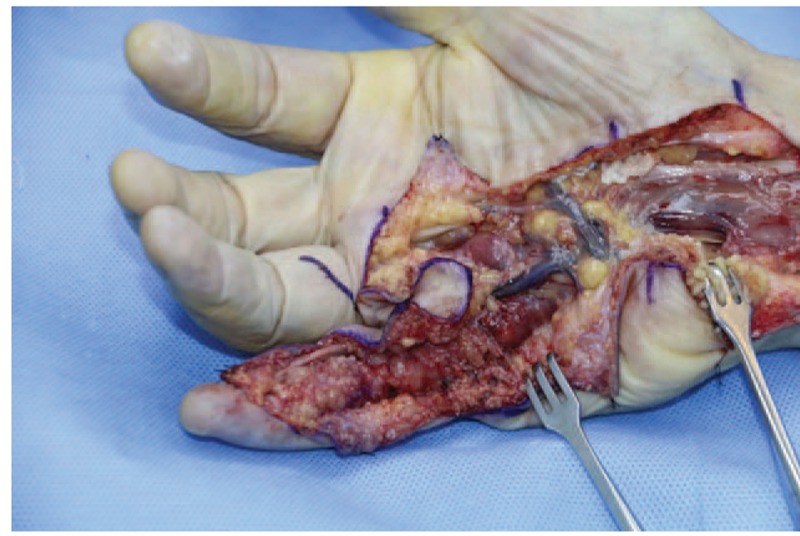
Radical debridement in area of palm.

**Figure 8:**
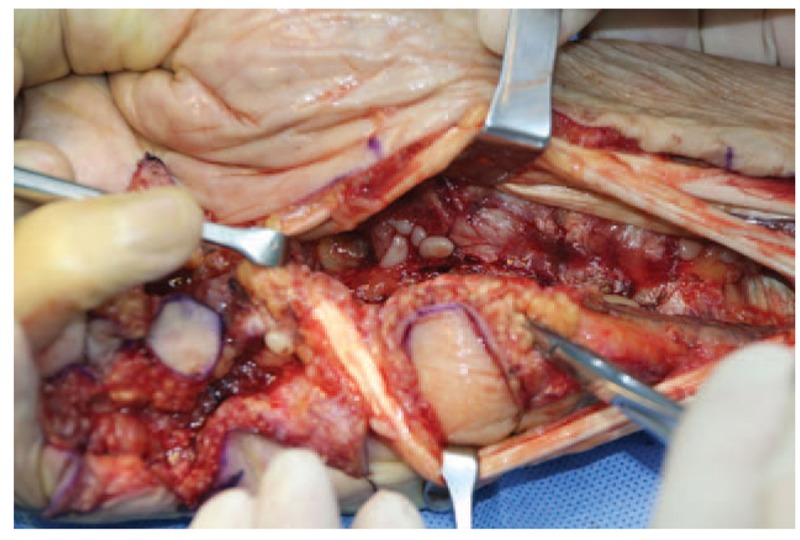
Radical debridement in area of wrist.

**Figure 9:**
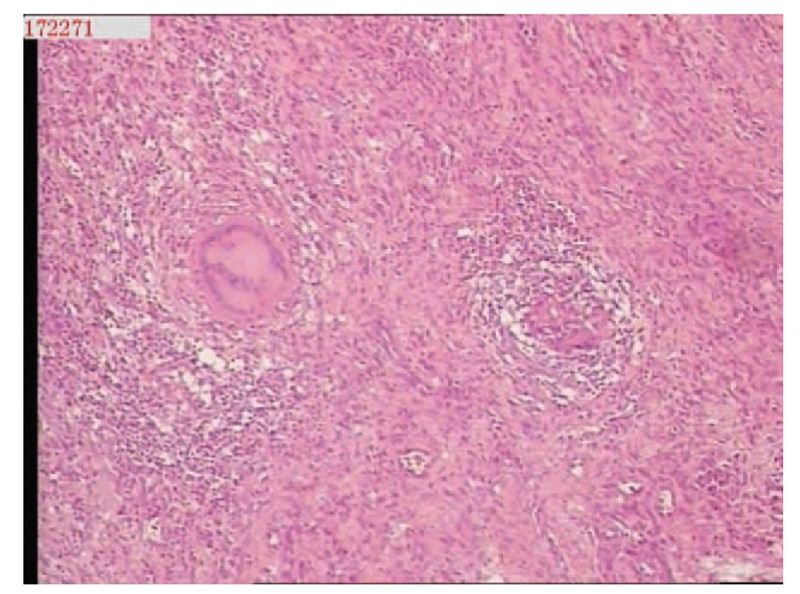
Pathological examination indicated *Mycobacterium tuberculosis* infection.

A 68-year-old adult man who had no history of tuberculosis (TB), had complained of pain and swelling in right palm and little finger for 3 months, as shown in Fig. 1. His body temperature ranged from 37 to 38 °C and symptoms such as pain, swelling, and movement limitation were generally non-specific. Hand x-ray just showed soft tissue swelling in little finger (Fig. 2). From Figs. 3 and 4, we could see extensive high signal in little finger, volar palm, and carpal tunnel according to T2-weighted image in MRI, indicating synovitis around flexor tendon. What's more, MRI showed nodular images in little finger, approximately sizing 5mm × 11 mm. Additionally, laboratory tests revealed C-reactive protein (CRP): 22 mg/L, erythrocyte sedimentation rate (ESR): 49 mm/h, and white blood cells (WBC): 11.8 × 10^9^/L. Considering mentioned above, aspiration biopsy guided by ultrasound was used as a key procedure before surgery. In Fig. 5, we inserted a needle into the swelling of palm to collect some soft tissue for pathological examination and result implied *Mycobacterium tuberculosis* (MTB) infection. Then, we performed radical synovectomy, as shown in Figs. 6–8. We could see multiple rice bodies mainly around flexor tendons in little finger, distributing in finger, volar palm, and carpal tunnel. We conducted pathological examination on some typical tissues and got the same result with previous one (Fig. 9). So, we convinced that final diagnosis was hand primary tuberculous synovitis. Afterwards, this patient was treated with antituberculous treatment. Postoperative body temperature ranged from 36 to 37 °C. One week after surgery, laboratory tests indicated CRP: 10 mg/L, ESR: 22 mm/h, and WBC: 7.8 × 10^9^/L. One year after surgery, pain and swelling of hand and wrist relieve and indicators of laboratory tests turn to normal showing CRP: 8 mg/L, ESR: 14 mm/h, and WBC: 7.2 × 10^9^/L.

## 4 Discussion

According to the “Global Tuberculosis (TB) 2015 Report” of the World Health Organization, about one-third of the world's population was infected by tuberculosis bacilli. Tuberculosis infection usually affects the respiratory system at first and then spreads extrapulmonary via lymphohematogenous route. As we known, the most common location is the vertebrae. It may also affect the pelvis, ankle, and wrist. Recently Bayram et al^[6]^ showed a rare case on wrist tenosynovitis infected by MTB which was diagnosed lately due to non-specific symptoms such as pain and swelling. Güner et al^[7]^ reported case series on wrist tenosynovitis infected by *Mycobacterium bovis*. Few reports on hand primary tuberculous synovitis had been published. Here, we presented a case that a 68-year-old man with pain and swelling in right palm and little finger for 3 months, shown in Fig. 1. Body temperature ranged from 37 to 38 °C. X-ray of hand presented soft tissue swelling of little finger and MRI showed synovitis around flexor tendon sheath of right thumb, little finger, and wrist (Figs. 2–4). The result of aspiration biopsy guided by ultrasound (Fig. 5) on 13rd Oct indicated MTB infection. Then we decided to operate radical debridement for that patient (Figs. 6–8). We collected some tissue for pathological examination and result showed MTB infection on 18th Oct (Fig. 9). Afterwards, the patient was treated with antituberculous treatment. One week after surgery, the CRP decreased from 22 to 10 mg/L, ESR from 49 to 22 mm/h, and WBC from 11.8 to 7.8 × 10^9^/L. One year after surgery, pain and swelling resolve. Hand primary tuberculous infection is a rare disease and it is tough to be diagnosed due to lack of special symptoms. The treatment of hand tuberculosis remains no consensus. Some authors^[8,9]^ reported that conservative treatment including chemotherapy, rehabilitation, and immobilization had successful clinical results. Some^[10,11]^ thought that surgical treatment alone without antituberculous chemotherapy was more likely to cause recurrence. While a comparable study between antituberculous chemotherapy and surgery–chemotherapy combination demonstrated no significant difference. In our case, we performed a successful treatment for hand tuberculous by radical synovectomy with antituberculous treatment. Up to now, pain and swelling of the hand have relieved and indicators of lab tests have turned to normal level. In spite of the satisfactory results, our treatment has some limitations. First, it needs a long term follow-up to assess the efficacy; second, this procedure may be too radical to effect functional recovery; third, we need more cases to evaluate this procedure.

In conclusion, hand primary tuberculous synovitis is rare. Few articles reported on this topic. It is easy to misdiagnosed, especially for patients without tuberculosis history and specific symptom. Tuberculosis infection should be kept in mind as an infectious agent when facing a case with unexplainable hand pain and swelling. Radical synovectomy with antituberculous treatment is an effective treatment, but we need further study to observe efficacy in a long term follow-up.
